# The Complexity of Nutritional Problems in Persons with Dementia: Expanding a Theoretical Model

**DOI:** 10.3233/JAD-230135

**Published:** 2023-10-24

**Authors:** Cornelia Pieternella van Buuren, Jenny Theodora van der Steen, Maria Olthof-Nefkens, Christian Bakker, Raymond Theodorus Catherina Maria Koopmans, Marieke Perry, Johanna Gezina Kalf

**Affiliations:** a Department of Primary and Community Care, Radboud University Medical Center, Nijmegen, The Netherlands; b ‘Joachim en Anna’, Center for Specialized Geriatric Care, Nijmegen, The Netherlands; c Radboudumc Alzheimer Center, Nijmegen, The Netherlands; d Department of Public Health and Primary Care, Leiden University Medical Center, Leiden, The Netherlands; e General Medical Practice, Velp, The Netherlands; f Groenhuysen, Center for Geriatric Care, Roosendaal, The Netherlands; g Department of Rehabilitation, Radboud University Medical Center, Donders Institute for Brain Cognition and Behaviour, Nijmegen, The Netherlands; h Zorggroep Maas & Waal, Beneden-Leeuwen, The Netherlands

**Keywords:** Alzheimer’s disease, dementia, feeding behavior, long-term care, nutritional disorders, theoretical model

## Abstract

**Background:**

Persons with dementia are at risk of developing nutritional problems. Theoretical models on nutritional problems have been developed, but have not been evaluated with healthcare professionals.

**Objective:**

This study aimed to explore the comprehensiveness and applicability of a theoretical model of nutritional problems in persons with dementia for daily nursing home practice.

**Methods:**

A qualitative design employing a combined deductive and inductive approach was used. Healthcare professionals were eligible to participate if they 1) had expert knowledge of and experience with nutritional problems related to dementia, and 2) worked in a nursing home affiliated with an academic network covering the east and south of the Netherlands. Three focus group interviews with 20 healthcare professionals from seven professions were held. We conducted thematic analysis and we compared themes with existing theoretical models from the literature.

**Results:**

We identified six themes, four of which corresponded with the existing models (observing and analysing nutritional problems; consequences of nutritional problems; functioning of the person with dementia; environmental factors). Interprofessional collaboration and ethical factors were identified as new themes. The analyses indicated interactions within each theme, between themes, and a bidirectional connection between themes.

**Conclusions:**

This study demonstrated the relevance of interprofessional collaboration and ethical considerations in nutritional problems related to dementia. It uncovered complex bidirectional relations within and between factors regarding nutritional problems. All aspects should be taken into account to minimize the consequences of nutritional problems for persons with dementia.

## INTRODUCTION

Nutritional problems are common in people with dementia, with prevalence rates from 30% to 60% [1, 2]. These problems refer to any problem occurring during eating and drinking that cause inadequate intake of fluids and food [3]. The consequences can be serious, such as malnutrition, dehydration, aspiration pneumonia, and even reduced social participation. Dehydration and aspiration pneumonia are two main causes of death in persons with dementia [4–6].

When identifying causes of nutritional problems in dementia, one could distinguish factors in several domains: physical, cognitive, and behavioral factors. The nature and severity of these factors varies between individuals, depending on the cause of the dementia and progression of the disease [7, 8]. Persons with dementia experience increased difficulties with chewing and swallowing, and apraxia in the more advanced stages of dementia in comparison with early stages [8, 9]. However, the motor and sensory components of swallowing are often overshadowed by cognitive and behavioral factors [7, 10]. Cognitive symptoms that limit effective nutritional intake are the inability to recognize food and reduced comprehension of language [10–12]. Other impairments include difficulty bringing food to the mouth due to apraxia or agnosia. Dental problems are an example of a physical factor that is known to affect nutritional intake [7, 13, 14]. Environmental aspects, such as the influence of social interaction and dining environment on eating and drinking are of influence on effective nutritional intake as well [7, 10, 12]. Wandering during mealtimes, apathy, or food refusal are behavioral symptoms that can result in inadequate intake of fluids and food [15].

Research has focused on single underlying causes of nutritional problems, but often lacks the context of multiple interrelated causes that are seen in clinical practice. Chang and Roberts developed a model to capture the problems and influencing aspects [14]. The model describes three aspects: 1) antecedents, such as perceptual deficits, cognitive impairment, and a lack of motor control, which influence 2) behavior, (nutritional problems). These problems can have 3) consequences, such as inadequate food intake, weight loss, or aspiration of food or liquid. Subsequently, this model was extended by Aselage, Amella and Watson by including contextual factors, such as the mealtime environment, mealtime patterns, and dyadic interaction [15]. However, both theoretical models were derived only from the literature and it is unclear whether their application in daily nursing home practice takes into account all relevant aspects in this context. This study aims to explore the comprehensiveness of these models related to their applicability in daily nursing home practice, considering experiences of professionals in daily care practice.

## METHODS

### Design

A qualitative design with focus groups using a combined inductive and deductive analytic approach was applied in this study. We followed the consolidated criteria for reporting qualitative research (completed COREQ checklist in the Supplementary Material) [16].

### Sample

Participants were purposefully sampled to represent a variety of relevant disciplines in each focus group session. Candidate participants were eligible to participate in the study if they met the inclusion criteria of 1) being experienced with dementia and dementia-related nutritional problems, and 2) working as a healthcare professional in a nursing home affiliated with the University Knowledge Network on Old Age Care in Nijmegen. Due to the multifactorial nature of the topic and to obtain a profound understanding of the topic, family caregivers were also invited when they met the first inclusion criterion. Family caregivers included close relatives, friends, and neighbors involved with the care. Participants were recruited by email and in person by the researcher EvB.

### Data collection

Data were collected during three face-to-face focus group sessions with healthcare professionals working in three different nursing homes between January and June 2019.

A topic list was used to guide the focus group discussions (Table 1), based on relevant literature, and the main themes of the existing theoretical models (e.g., behavior/nutritional problems, causes/antecedents, and consequences) [14, 17]. All focus group discussions started with an introduction by the facilitator (EvB), observer (occupational therapy student), and participants, followed by a discussion guided by the topic list. The observer made field notes during each discussion. EvB was working as a speech and language therapist at the time of the study. She clarified her role as a researcher at the start of the sessions as she was known to several participants, as some of them were colleagues. The focus group discussions were video recorded, for which the participants provided their written consent. A copy of the informed consent and certificate of attendance was offered to the participants. The topic list (Table 1) covered six open questions for which we deemed pilot-testing with healthcare professionals prior to data collection unnecessary.

**Table 1:** Topic list

Questions	Prompts
**1.** What eating and drinking problems do you come across in your practice?	*What exactly do you think these nutritional problems are?*
	*Can you give examples?*
	*Can you describe an example situation?*
	*What causes have you identified?*
**2.** What consequences do eating and drinking problems have?	*How do you know that?*
	*Can you give examples?*
**3.** What is your role in eating and drinking problems?	*What exactly do you do?*
	*How?*
**4.** When are you called in?	*Who calls you in?*
	*How do you feel about the timing?*
**5.** Do you ever collaborate with someone else when it comes to eating and drinking problems?	*Who do you work with?*
	*Why do you collaborate with them?*
	*How does that work?*
	*How often do you work together?*
**6.** What issues have we not yet discussed?

### Data analysis

Data were transcribed verbatim by the researcher EvB. Thematic analysis was supported using Atlas.ti 8 [18]. Data were analyzed by the researcher EvB, trained in the basics of conducting qualitative research while the research team’s expertise covered extensive qualitative and quantitative research. EvB coded small units. Through this open coding process, new codes were identified in an inductive way, and discussed by the researchers EvB, MO, and HK [18, 19]. Subsequently, the codes were compared to the themes of the existing models, to secure the deductive part of data analysis. After three rounds of data collection and analysis, an adapted model was developed by the researcher EvB based on the codes and themes that were identified during data-analysis. The existing theoretical model [14] was complemented with the findings from this study. The connections between the themes in the model were discussed with the participants of the focus group sessions and research team, and adapted in the model. The model was discussed in phases, first with HK and MO, both of whom are researchers with experience in qualitative research and backgrounds in speech and language therapy. Subsequently, the model was presented to all participants of the three focus group sessions by individual email and adjusted based on their feedback to offer member checking and create a final model. Next, we involved additional researchers with specific expertise and various backgrounds to support the quality of the study. The adaptation of the model was discussed with MP and JvdS, both experienced dementia care researchers with backgrounds as a general practitioner and methodologist, respectively. The three researchers discussed the themes on code level to examine the content and relationships between the themes. Finally, the model was reviewed by RK and CB, who are experienced dementia researchers with a background in elderly care medicine and psychology in dementia, while RK and JvdS are also experts in palliative care.

### Ethical considerations

This study was conducted according to the principles of the Declaration of Helsinki (version 13) [20]. The study was declared exempt from the Medical Research Involving Human Subjects Act by the research ethics committee of the Radboud university medical center Nijmegen (file number CMO: 2019-5139).

## RESULTS

A total of 20 healthcare professionals (19 women) participated in three focus group sessions, with participants from seven professions employed by three nursing homes. No family caregivers were willing to participate. The focus group discussions lasted 60 to 90 min, and took place in a conference room at one of the nursing homes. Five participants responded by e-mail to the member checking invitation. Details of the participants are shown in Table 2.

**Table 2:** Participant characteristics

	Focus group 1	Focus group 2	Focus group 3
N=20	N=6	N=7	N=7
Gender	∼
Men	1	0	0
Women	5	7	7
Profession	∼	∼	∼
Speech and language therapist	1	2	2
Occupational therapist	1	1	2
Dietitian	1	1	1
Psychologist	1	1	0
Cognitive rehabilitation therapist	0	1	1
Nurse	1	1	0
Elderly care physician	1	0	1
Total	6	7	7

Thematic analysis resulted in six themes capturing nutrional problems and related factors: 1) observing and analyzing nutritional problems; 2) consequences of nutritional problems; 3) functioning of the person with dementia; 4) interprofessional collaboration; 5) ethical factors; and 6) environmental factors. Below, we describe the six themes, after which we detail the complex nature of the relationships between the themes.

*1. Observing and analyzing nutritional problems*

The participants reported having witnessed various problems during mealtimes regarding the ability to get food into the mouth, such as an inability to recognize or use cutlery or food.

“*The way that they handle cutlery is not what it was and, ehm, people no longer know how to bring the food to their mouth (* ...  *). So I think that handling cutlery is already a basis, so to speak. And that can be strength, or cognitive.*” *(cognitive rehabilitation therapist 1)*

An inability to chew and swallow were indicated as frequent problems. Coughing due to aspiration during eating or drinking was referred to as the most frequent and severe problem in persons with dementia. Moreover, suffering from a lack of proper dentures or pain in the mouth were mentioned as reducing chewing ability, making eating bothersome or painful. Finally, an altered sense of hunger and thirst was referred to as a problem. A person with dementia may experience not feeling hungry, and therefore they might refuse to eat or only eat a small portion.

“*The stimulus is no longer transmitted properly either. So, ehm, there is no feeling of hunger or thirst sometimes.*” *(dietitian 2)*

The participants identified adequate detection of nutritional problems as essential. Analyzing the problems, searching for the underlying causes of eating and drinking problems was described as a shared task and was sometimes experienced as challenging. It was mentioned that the consequences of eating and drinking problems are observed at times, while the underlying causes are not always detected. Moreover, multiple nutritional problems may interact, such as oropharyngeal dysphagia and apraxia. Combinations of problems were mentioned as being challenging and call for alertness of all personsinvolved.

*2. Consequences of nutritional problems*

Aspiration pneumonia was mentioned as a serious consequence of swallowing problems. Further, the participants stated that consequences may be underestimated. Persons may lose weight gradually over a longer period, which may go largely unnoticed when not weighed regularly, so it may look like a less serious problem than is actually the case.

“*What we sometimes see is that, ehm, the progression of weight loss. Well, if that is really measured over six months, and someone has lost, say half a kilogram every time, then they often look at the last weight and say* “*oh, that’s actually not that much*” (...  *) Then you sometimes see that it can be ten kilograms in six months and then it’s rather late in the day.*” *(dietitian 3)*

A decline in mobility and cognition were considered as consequences that may occur when there is a reduced intake of food and fluids. Social aspects of eating and drinking – such as less enjoyment at mealtimes or reduced acceptance by others – may result in an overall decline in the quality of life experienced according to some participants.

“*The social aspect, where one realises that things are not going so well anymore, that shame also plays a role so that quality of life decreases*” *(occupational therapist 3)*

When weight loss is not detected in an early stage, it can be difficult to start an effective intervention and stabilize the weight loss, in particular when a person with dementia has less energy, resulting in an insufficient amount of daily nutrition. In this case, participants identified a vicious circle, because eating requires energy, and may become increasingly difficult.

*3. Functioning of the person with dementia:*

Agitation, anxiety, and mood were mentioned as factors affecting eating and drinking.

“*That they are fed, for example, and it goes too fast, right, the feeling that they have no control. You can become anxious*”*. (speech and language therapist 4)*

The interpretation of mental and cognitive aspects, such as the influence of depression on the intake of nutrition, was experienced as difficult by most participants. Additionally, agitation was frequently observed, in terms of walking away from the table at mealtimes. Being distracted was the most frequently reported cognitive problem. A relationship between distraction during mealtimes and a lower intake of food and fluids was suggested by some participants. Reduced initiative was also expected to be strongly associated with an inadequate intake of nutrition.

“*People sometimes forget that they*’*ve eaten.*” *(speech and language therapist 5)*

Some participants considered physical problems, such as incontinence or urinary tract infection, to influence the intake of nutrition, in terms of not feeling well or walking away from the table. It was mentioned that medication can cause drowsiness, resulting in reduced focus on a meal or (almost) falling asleep, and side effects on intake being underestimated. Pain and feeling sick were also recognized as factors that could influence food intake.

*4. Interprofessional collaboration*

The participants indicated that often no one has a sufficient overview regarding the development of problems or the overall daily nutritional intake of persons with dementia. It was questioned whether detecting nutritional problems should be the task of nurses only. Some participants felt that every team member should be a stakeholder regarding the detection and management of nutritional problems. Analyzing nutritional problems and subsequently considering consulting a healthcare professional, such as a speech and language therapist or occupational therapist, was pointed out as crucial.

Detection and collaboration between nurses and other healthcare professionals was an important topic of discussion. Nutritional problems are not always detected, due to insufficient knowledge of what these problems are or which healthcare professional to consult. The consequences of nutritional problems were sometimes seen in practice, but not always linked to the appropriate cause(s), resulting in inadequate or late consultation of healthcare professionals. In addition, a lack of continuity at care units – in the sense of increased presence of flex workers – was referred to as a limitation in adequately detecting problems.

“*Well, it all starts with the person observing, doesn*’*t it? If you have a good permanent team then, ehm, with people who know the residents, yes, then things go well. If you come to a facility with a lot of workers with flexible contracts, then, ehm, where there is a lot of alternation in care... Yes, then I think you can make improvements there.*” *(occupational therapist 1)*

The motivation of a team to provide good care of a person with dementia was experienced to influence the way the team handles nutritional issues.

“*What kind of team do you have. Because you also have teams that stimulate each other a lot and* ...   “*come on, let*’*s get cracking*”, “*we*’*re going to take good care of this resident*”*, and* “*let*’*s go*” ...   *yes, those are much more motivated than the others who can*’*t be bothered.*” *(psychologist 1)*

*5. Ethical considerations*

Some participants said that opinions on nutritional intake determine the detection and management of nutritional problems in practice, because teams can struggle with handling problems in case of different norms and values of family members.

“*That can be very difficult at times. I*’*ve organised a moral deliberation for such a resident at times, to take a good look and see, like, what are our values and standards and those of the family. On the one hand, you want to allow them room for their own standards and values, but also set a limit somewhere regarding what is acceptable for the resident’s safety. So you discuss all those perspectives together.*” *(elderly care physician 2)*

Accepting that safe and efficient eating and drinking is problematic or nearly impossible, was appointed by particpants to result in a struggle for both healthcare professionals and family. They experienced that sometimes persons with dementia have a limited freedom of choice about what to eat and where to eat, and preferences of persons with dementia are ignored.

“*If he has a cheese sandwich once, he is always offered cheese sandwiches, even though he may want ham. I think that choice is important too.*” *(nurse 1)*

Religion was found to influence decision making and agreement between relatives of persons with dementia and healthcare professionals. Finding the balance between being professional and showing respect towards religion was considered to hold importance.

“*It’s even more important for families from a different background from a religious point of view, like, we must have done everything and then it*’*s in the hands of God or Allah. You must feel that they have tried everything, but they sometimes cross certain boundaries of the care unit or certain caregivers in this.*” *(elderly care physician 2)*

In advanced stages of dementia, nutritional intake may be very limited or even become impossible or unsafe. At such a critical point, decision making is regarded a collaborative task. Differences in norms and values regarding eating and drinking were said to influence decision making, and sometimes result in moral dilemmas. In such situations, it was considered important to keep an open dialogue to reach a mutual decision.

“*The context allows family to say that it suits him to keep his mouth shut right now and stop eating and drinking, followed by a consensus to accept the behavior. Another family may say* “*he was never like that*”*, then he probably, ehm, won’t have had breakfast or maybe he’s afraid or, ehm, he has a rotten tooth. In fact, you have to come to some kind of consensus about what actually suits this person well.*” *(elderly care physician 1)*

*6. Environmental factors*

Persons with dementia may have difficulty recognizing modern food or utensils in contrast to, for example, traditional china or other dinnerware that they may know from their past. The use of modern food or utensils can therefore be an influencing factor that may limit their intake of food and fluids during mealtimes. Noise during mealtimes was believed to increase agitation and noise at the care unit was stated as an issue that could be improved.

“*Sometimes it seems as if a construction worker is at work, loud music, visitors who come in anyway, one joins the meal, the other doesn*’*t. The walkers among the residents. The unrest. The telephone. Other disciplines coming in during dinner.*” *(psychologist 1)*

The position when seated at mealtimes was discussed as another environmental factor. The accessibility of a plate or a glass is sometimes hindered when sitting too far away from the table.

“*The positioning. Not just in terms of who sits opposite whom at the table. Is someone sitting at the correct table height? Is he sitting comfortably in his chair or wheelchair? I regularly visit people whose wheelchair is slightly tilted, whereby they sit with their knees against the table top and cannot reach their food.*” *(occupational therapist 1)*

The social environment, such as having a meal and activities at the same table, was thought to have an influence on eating and drinking.

“*If you sit at that dining table all day, right, for coffee and everything, then it’s kind of your hangout. But then it*’*s not very clear what your starting moment is, like what*’*s the intention of the activity? Whereas when you*’*re away from the table, the table is set, like,* “*come, Mrs Smith, we*’*re going to eat*” *and everything*’*s already there. Then it*’*s immediately clear, because it won’t take too long before you give a helping hand. And there*’*s the smell, then you are much further in habituating, like,* “*oh, I understand what the intention is here*”.” *(occupational therapist 3)*

Additionally, the influence of the person assisting the persons with dementia was frequently mentioned. Nurses can have limited time, resulting in a hasty offering of food. This may lead to an increased risk of aspiration for persons with swallowing problems. Moreover, eating alone in a room or amidst a group was mentioned as an environmental factor. Given that short timeframes for daily nutritional intake exist at nursing home care units, persons with dementia may lose their appetite and not want another meal, causing a limited nutritional intake.

“*That people don’t get out of bed until eleven o’clock and then eat breakfast and maybe start another meal an hour or an hour and a half later. But people who go to bed again very early, just after the evening meal, so that they don*’*t eat anything in the evening. So that time of eating is often quite limited, which reduces the intake.*” *(dietitian 1)*

Ordering of meals with an adapted consistency was mentioned as challenging at weekends, due to financial cuts in the organization or policy, because disciplines such as speech language therapists or dietitians are absent at weekends.

“*When you go to the kitchen on a Saturday morning, like, we have this and this admission, it*’*s not always easy to get that arranged without the advice of a speech therapist or dietician.*” *(nurse 2)*

One has to deal with the supplies of the organization. For instance, the kitchen of the facility does not provide hot meals at different times. Furthermore, a limited selection of food is available for people diagnosed with oropharyngeal dysphagia.

“*If you now said, for example, like,* “*I want to have my hot meal at 5 PM or served at 5.30 PM*”*, then, well, no, that*’*s not possible because as a kitchen we have been set up so that it is ready at 12. How can you then respond to the client*’*s wishes and how do you facilitate that?*” *(psychologist 1)*

### Proposed adaptation of the theoretical model

**Fig. 1:**
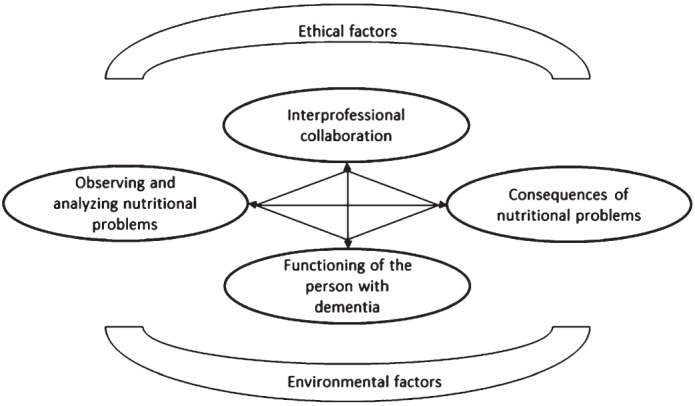
Proposed model of nutritional problems in persons with dementia applicable in daily nursing home practice. It includes the two new themes interprofessional collaboration and ethical factors, and a bidirectional link between the themes.

We found complex interactions within each of the aforementioned themes. Further, connections between themes add to the complexity of dementia-related nutritional problems. Based on these findings, we adapted the existing model, showing the relationship between themes (Fig. 1). The management of nutritional problems requires interprofessional collaboration, because it is advisable to identify underlying causes in order to start appropriate interventions and prevent or minimize the consequences of nutritional problems. Regarding swallowing problems, the nature and severity of swallowing should be examined by clinical observations, taking the influence of cognitive and behavioral factors into account [7, 21]. Moreover, a mutual understanding of tasks and coordination of addressing nutritional problems is warranted in clinical practice. Ethical factors in turn influence interprofessional collaboration, each team member having their own norms and values and cultural background. These differences affect the interpretation and management of nutritional problems. Finally, we consider a third level of complexity. Analyzing and discussing the themes and codes, different from a unidirectional relationship between themes as displayed in the existing model, we found a bidirectional relationship between observing and analyzing nutritional problems (theme 1) and consequences of nutritional problems (theme 2). Moreover, we found bidirectional relationships between other themes as well. We illustrate the multifactorial nature of nutritional problems resulting in a vicious circle using an example:

Behavioral problems such as food refusal (functioning of the person with dementia) leads to limited or no intake (observing and analyzing nutritional problems). This may result in weight loss and subsequent malnutrition (consequences of nutritional problems), which makes the person with dementia feel sick or weak (functioning of the person with dementia), further leading to increased weight loss (consequences of nutritional problems) or difficulty swallowing (requiring reobserving- and analyzing problems) that may increase the risk of developing aspiration pneumonia (consequences of nutritional problems) when taking action is delayed.

Further, ethical and environmental factors influence nutritional problems more indirectly in comparison to the other themes. Moral dilemmas may occur in decision-making on diagnostics and treatment in advanced dementia, when a vicious circle and subsequent deterioration appears. In these situations, decision making can be difficult. Environmental factors, such as the position of a person and the social environment during mealtimes influence nutritional problems as well, but are more distantly related to the themes in the middle of the model which relate directly to what can be seen and to the persons involved.

## DISCUSSION

We adapted an existing model comprising four themes regarding nutritional problems, adding two new aspects: interprofessional collaboration and ethical factors [14, 15]. As such, this study builds a bridge between the theory around nutritional problems in persons with dementia and daily clinical practice in nursing homes. The other four themes were confirmed in our study by professionals from clinical practice [14, 15]; observing and analyzing nutritional problems, consequences of nutritional problems, functioning of the person with dementia, and environmental factors. Beyond these aspects, connections within each theme, between themes, and a bidirectional connection between the themes were identified. This differs from previous models, which describe a more linear connection. Environmental factors influence mealtimes and are therefore most strongly related to functioning of the person with dementia. Interprofessional collaboration and ethical factors are related to the other themes in the model, but mainly to observing and analyzing nutritional problems and minimizing consequences of nutritional problems. The relation between these two themes and functioning of the person with dementia and environmental factors is more indirect, which explains the position in the model. Ethical factors, such as norms and values of healthcare professionals, influence interprofessional collaboration. Additionally, collaboration of the multidisciplinary team influences the timing of observation and analysis. In case of late timing, for example, a person may have lost a lot of weight and therefore experience more severe consequences. These new findings underline the complexity of nutritional problems in daily clinical practice.

The newly added theme of ethical factors was found before to influence the way nutritional problems are handled, not only by healthcare professionals, but by relatives of persons with dementia as well [15]. Norms and values are known to differ per person and are of influence on situations around eating and drinking [3]. Persons with advanced dementia depend on decisions taken by healthcare professionals and relatives, because their own decision making capacity is impaired [22, 23]. Decision making is considered a struggle for relatives and professionals involved when nutritional intake becomes (nearly) impossible or unsafe, especially when norms and values regarding end-of-life-decisions differ [24]. Such end-of-life decisions can be difficult and complex, and should be made as a team and family together with respect towards each other despite possible differences [24–27]. In these situations, artificial nutrition and hydration may be considered. However, because observational studies indicate that enteral feeding does not prolong life or improve a person with dementia’s quality of life [28–30], this is rarely applied in the Netherlands.

Interprofessional collaboration as a relevant influencing factor was identified as a novel theme as well. In the Netherlands, it is common to work as a team on care units in a nursing home, with nursing staff being the heart of the professional team, supplemented with other healthcare professionals. In most nursing homes, all healthcare professionals, including an elderly care physician as primary practitioner, are employed by the nursing home [31]. Although interprofessional collaboration is considered a very important aspect of nursing home care, our study showed that detecting nutritional problems as a team was identified as challenging, referring to a delay in consulting the right expert. However, interprofessional collaboration is necessary to provide adequate patient care, especially in complex cases such as nutritional problems [32, 33]. Moreover, our finding that insufficient knowledge of the problems can influence problem analysis and subsequent consultation of the right healthcare professional, was confirmed in earlier qualitative research [34]. Another factor that we found to contribute to late detection is the increased use of temporary flex workers to compensate for staff shortages at care units. This underlines the importance of a stable team of professionals who know their residents and are therefore able to timely detect subtle changes and problems. None of the healthcare professionals involved is present all the time, so communication and task division were considered very important by the participants of our study. This is also discussed by Sloane et al. [35], who describe inadequate staffing at care units, which can be linked to logistic limitations.

The results of this study should be interpreted taking its strengths and limitations into consideration. The main strength of this study is the contrasting design compared to other studies, that investigated nutritional problems in persons with dementia by reviewing the literature only, rather than being informed by those working in clinical practice [14, 15]. We consider reaching theoretical and inductive thematic saturation, because we identified no new codes and themes during the third focus group discussion and codes regarding the new themes (interprofessional collaboration and ethical factors) were mentioned in each focus group discussion. Moreover, the content of these new themes corresponds with what is known from recent literature about multidisciplinary collaboration more generally [36, 37]. We applied the following important qualitative research quality criteria. We performed a member check to support the validity of the study results by sending a brief report to all participants after each focus group for feedback. Moreover, multiple researchers with different research and clinical backgrounds (investigator triangulation) were involved in the data analysis and development of the model to promote credibility.

A potential limitation of this study is possible researchers’ bias. The researcher who led the focus group discussions (EvB) was known to several participants as some of them were colleagues. Thus, participants might have been reluctant to share all of their experiences due to this existing professional relationship and knowing EvB was working as a speech and language therapist. However, on the positive side this might have promoted a sense of safety for the participants, encouraging them not to hold back on sharing what they wanted. Another limitation of this study is the absence of family caregivers, despite the fact that they were invited to participate. Family caregivers might have described nutritional problems from another perspective.

### Future perspectives

This study adds further support to the view that all aspects related to nutritional problems in dementia should be considered when assessing and subsequently minimizing its consequences. It may be challenging for case managers and coordinators to keep all team members aware and alert of the complexity of this issue. Also, the growing number of people with dementia [38] requires optimal care models and future research is awaited to demonstrate how nutritional problems in dementia can be managed in the best way possible.

## Supplementary Material

Supplementary Material

## Data Availability

The data supporting the findings of this study are available on request from the corresponding author. The data are not publicly available due to privacy or ethical restrictions.

## References

[1] Li L , Zhao Y , Wang Y , Wang Z (2020) Overview of systematic reviews: Effectiveness of non-pharmacological interventions for eating difficulties in people with dementia. J Adv Nurs 76, 2830–2848. 32852131 10.1111/jan.14492

[2] Mitchell SL , Teno JM , Kiely DK , Shaffer ML , Jones RN , Prigerson HG , Volicer L , Givens JL , Hamel MB (2009) The clinical course of advanced dementia. N Engl J Med 361, 1529–1538. 19828530 10.1056/NEJMoa0902234 PMC2778850

[3] Volkert D , Chourdakis M , Faxen-Irving G , Fruhwald T , Landi F , Suominen MH , Vandewoude M , Wirth R , Schneider SM (2015) ESPEN guidelines on nutrition in dementia. Clin Nutr 34, 1052–1073. 26522922 10.1016/j.clnu.2015.09.004

[4] Wojszel ZB (2020) Impending low intake dehydration at admission to a geriatric ward- prevalence and correlates in a cross-sectional study. Nutrients 12, 398. 32024303 10.3390/nu12020398 PMC7071250

[5] Manabe T , Fujikura Y , Mizukami K , Akatsu H , Kudo K (2019) Pneumonia-associated death in patients with dementia: A systematic review and meta-analysis. PLoS One 14, 1–14. 10.1371/journal.pone.0213825 PMC6417730 30870526

[6] Todd S , Barr S , Passmore AP (2013) Cause of death in Alzheimer’s disease: A cohort study. QJM 106, 747–753. 23653484 10.1093/qjmed/hct103

[7] Rogus-Pulia N , Malandraki GA , Johnson S , Robbins J (2015) Understanding dysphagia in dementia: The present and the future. Cur Phys Med Rehabil Rep 3, 86–97.

[8] Miarons M , Clave P , Wijngaard R , Ortega O , Arreola V , Nascimento W , Rofes L (2018) Pathophysiology of oropharyngeal dysphagia assessed by videofluoroscopy in patients with dementia taking antipsychotics. J Am Med Dir Assoc 19, 812.e1–812. e10. 10.1016/j.jamda.2018.04.016 30149844

[9] Borders JC , Blanke S , Johnson S , Gilmore-Bykovskyi A , Rogus-Pulia N (2020) Efficacy of mealtime interventions for malnutrition and oral intake in persons with dementia: A systematic review. Alzheimer Dis Assoc Disord 34, 366–379. 32530831 10.1097/WAD.0000000000000387 PMC7679285

[10] Alagiakrishnan K , Bhanji RA , Kurian M (2013) Evaluation and management of oropharyngeal dysphagia in different types of dementia: A systematic review. Arch Gerontol Geriatr 56, 1–9. 22608838 10.1016/j.archger.2012.04.011

[11] Easterling CS , Robbins E (2008) Dementia and dysphagia. Geriatr Nurs 29, 275–285. 18694703 10.1016/j.gerinurse.2007.10.015

[12] Nogueira D , Reis E (2013) Swallowing disorders in nursing home residents: How can the problem be explained? Clin Interv Aging 8, 221–227. 23449951 10.2147/CIA.S39452 PMC3581290

[13] Murphy JL , Holmes J , Brooks C (2017) Nutrition and dementia care: Developing an evidence-based model for nutritional care in nursing homes. BMC Geriatr 17, 1–14. 28196475 10.1186/s12877-017-0443-2 PMC5309970

[14] Chang CC , Roberts BL (2008) Feeding difficulty in older adults with dementia. J Clin Nurs 17, 2266–2274. 18705703 10.1111/j.1365-2702.2007.02275.x

[15] Aselage MB , Amella EJ (2010) An evolutionary analysis of mealtime difficulties in older adults with dementia. J Clin Nurs 19, 33–41. 20500242 10.1111/j.1365-2702.2009.02969.x

[16] Tong AS , P. , Craig , J. (2007) Consolidated criteria for reporting qualitative research (COREQ): A 32-item checklist for interviews and focus groups. Int J Qual Health Care 19, 349–357. 17872937 10.1093/intqhc/mzm042

[17] Aselage MB , Amella EJ , Watson R (2011) State of the science: Alleviating mealtime difficulties in nursing home residents with dementia. Nurs Outlook 59, 210–214. 21757077 10.1016/j.outlook.2011.05.009

[18] Braun V , Clarke V (2012) Thematic analysis. In APA handbook of research methods in psychology, Vol 2: Research designs: Quantitative, qualitative, neuropsychological, and biological, Cooper H, Camic PM, Long DL, Panter AT, Rindskopf D, Sher KJ, eds. American Psychological Association, Washington, pp. 57–71.

[19] Holloway I , Wheeler S (2010) Qualitative Research in Nursing and Healthcare, 3rd edition, Wiley-Blackwell Chichester, United Kingdom.

[20] World Medical Association (2013) World Medical Association Declaration of Helsinki: Ethical principles for medical research involving human subjects. JAMA 310, 2191–2194. 24141714 10.1001/jama.2013.281053

[21] Gilmore-Bykovskyi AL , Rogus-Pulia N (2018) Temporal associations between caregiving approach, behavioral symptoms and observable indicators of aspiration in nursing home residents with dementia. J Nutr Health Aging 22, 400–406. 29484354 10.1007/s12603-017-0943-y PMC5830143

[22] Matarasso Greenfeld S , Gil E , Agmon M (2022) A bridge to cross: Tube feeding and the barriers to implementation of palliative care for the advanced dementia patient. J Clin Nurs 31, 1826–1834. 32734659 10.1111/jocn.15437

[23] Heuberger R , Wong H (2019) Knowledge, attitudes, and beliefs of physicians and other health care providers regarding artificial nutrition and hydration at the end of life. J Aging Health 31, 1121–1133. 29519177 10.1177/0898264318762850

[24] Barrado-Martin Y , Hatter L , Moore KJ , Sampson EL , Rait G , Manthorpe J , Smith CH , Nair P , Davies N (2021) Nutrition and hydration for people living with dementia near the end of life: A qualitative systematic review. J Adv Nurs 77, 664–680. 33249602 10.1111/jan.14654 PMC7898342

[25] Eisenmann Y , Golla H , Schmidt H , Voltz R , Perrar KM (2020) Palliative care in advanced dementia. Front Psychiatry 11, 699. 32792997 10.3389/fpsyt.2020.00699 PMC7394698

[26] Ng AYM , Takemura N , Xu X , Smith R , Kwok JY , Cheung DST , Lin CC (2022) The effects of advance care planning intervention on nursing home residents: A systematic review and meta-analysis of randomised controlled trials. Int J Nurs Stud 132, 104276. 35667145 10.1016/j.ijnurstu.2022.104276

[27] Bolt SR , van der Steen JT , Khemai C , Schols J , Zwakhalen SMG , Meijers JMM (2022) The perspectives of people with dementia on their future, end of life and on being cared for by others: A qualitative study. J Clin Nurs 31, 1738–1752. 33432696 10.1111/jocn.15644 PMC9290953

[28] Hanson LC , Ersek M , Gilliam R , Carey TS (2011) Oral feeding options for people with dementia: A systematic review. J Am Geriatr Soc 59, 463–472. 21391936 10.1111/j.1532-5415.2011.03320.x PMC3164780

[29] Schneider PL , Fruchtman C , Indenbaum J , Neuman E , Wilson C , Keville T (2021) Ethical considerations concerning use of percutaneous endoscopic gastrostomy feeding tubes in patients with advanced dementia. Perm J 25, 20.302. 10.7812/TPP/20.302 PMC8817934 35348076

[30] Davies N , Barrado-Martin Y , Vickerstaff V , Rait G , Fukui A , Candy B , Smith CH , Manthorpe J , Moore KJ , Sampson EL (2021) Enteral tube feeding for people with severe dementia. Cochrane Database Syst Rev 8, 1–94. 10.1002/14651858.CD013503.pub2 PMC8407048 34387363

[31] Koopmans R , Pellegrom M , van der Geer ER (2017) The Dutch move beyond the concept of nursing home physician specialists. J Am Med Dir Assoc 18, 746–749. 28668662 10.1016/j.jamda.2017.05.013

[32] Allan CM , Campbell WN , Guptill CA , Stephenson FF , Campbell KE (2006) A conceptual model for interprofessional education: The international classification of functioning, disability and health (ICF). J Interprof Care 20, 235–245. 16777791 10.1080/13561820600718139

[33] Tanaka M (2003) Multidisciplinary team approach for elderly patients. Geriatr Gerontol Int 3, 69–72.

[34] Lea EJ , Goldberg LR , Price AD , Tierney LT , McInerney F (2017) Staff awareness of food and fluid care needs for older people with dementia in residential care: A qualitative study. J Clin Nurs 26, 5169–5178. 28880410 10.1111/jocn.14066

[35] Sloane PD , Ivey J , Helton M , Barrick AL , Cerna A (2008) Nutritional issues in long-term care. J Am Med Dir Assoc 9, 476–485. 18755420 10.1016/j.jamda.2008.03.005

[36] Doornebosch AJ , Smaling HJA , Achterberg WP (2022) Interprofessional collaboration in long-term care and rehabilitation: A systematic review. J Am Med Dir Assoc 23, 764–777. 35065048 10.1016/j.jamda.2021.12.028

[37] Schwartz DB , Barrocas A , Annetta MG , Stratton K , McGinnis C , Hardy G , Wong T , Arenas D , Turon-Findley MP , Kliger RG , Corkins KG , Mirtallo J , Amagai T , Guenter P ; ASPEN International Clinical Ethics Position Paper Update Workgroup (2021) Ethical aspects of artificially administered nutrition and hydration: An ASPEN position paper. Nutr Clin Pract 36, 254–267. 33616284 10.1002/ncp.10633

[38] GBD 2019 Dementia Forecasting Collaborators (2022) Estimation of the global prevalence of dementia in 2019 and forecasted prevalence in 2050: An analysis for the Global Burden of Disease Study 2019. Lancet Public Health 7, e105–e125. 34998485 10.1016/S2468-2667(21)00249-8 PMC8810394

